# An analysis of variability in genome organisation of intracellular calcium release channels across insect orders

**DOI:** 10.1016/j.gene.2018.05.075

**Published:** 2018-09-05

**Authors:** Bartlomiej J. Troczka, Ewan Richardson, Rafael A. Homem, T.G. Emyr Davies

**Affiliations:** Biointeractions and Crop Protection Department, Rothamsted Research, Harpenden AL5 2JQ, UK

**Keywords:** ORF, open reading frame, RyR, ryanodine receptor, IP_3_R, inositol 1,4,5-trisphosphate receptor, RT-PCR, reverse transcription polymerase chain reaction, TM, trans-membrane, Ryanodine receptor, Inositol 1,4,5-trisphosphate receptor, Insect

## Abstract

Using publicly available genomic data, combined with RT-PCR validation, we explore structural genomic variation for two major ion channels across insect classes. We have manually curated ryanodine receptor (RyR) and inositol 1,4,5-trisphosphate receptor (IP_3_R) ORFs and their corresponding genomic structures from 26 different insects covering major insect orders. We found that, despite high protein identity for both RyRs (>75%) and IP_3_Rs (~67%), the overall complexity of the gene structure varies greatly between different insect orders with the simplest genes (fewest introns) found in Diptera and the most complex in Lepidoptera. Analysis of intron conservation patterns indicated that the majority of conserved introns are found close to the 5′ end of the channels and in RyR around the highly conserved mutually exclusive splice site. Of the two channels the IP_3_Rs appear to have a less well conserved organisation with a greater overall number of unique introns seen between insect orders. We experimentally validated two of the manually curated ORFs for IP_3_Rs and confirmed an atypical (3799aa) IP_3_R receptor in *Myzus persicae*, which is approximately 1000 amino acids larger than previously reported for IP_3_Rs.

## Highlights

• Mapped the genomic structures of RyR and IP_3_R from 26 different insect species
• Overall complexity of RyR and IP_3_R gene structure varies greatly between different insect orders.
• Identified and experimentally validated an atypically large IP_3_R receptor in aphid species

## 1 Introduction

Recent advances in sequencing technologies have led to a rapid increase in the number of publicly available genomes, including those of insects. The insect genomes have been found to be incredibly diverse in size; the smallest genome, that of *Belgica antarctica*, comprising only 99 megabases (Kelley et al., 2014) whilst the ~6.5 gigabase genome of *Locusta migratoria* is the largest genome within the animal kingdom (Wang et al., 2014). This diversity in genome size is also reflected in gene architecture complexity; in other words, despite a high degree of similarity at the protein level, the genomic architecture of genes can vary greatly between insect orders. Recent genome annotation efforts suggest that insect ryanodine receptors (RyRs) are a classic example of this type of complexity e.g. the number of exons present within this gene can vary from 26 in the fruit fly *D. melanogaster* (Takeshima et al., 1994) to 55 in the red flour beetle *T. castaneum* (Liu et al., 2014) and 98 in aphids (e.g. *Myzus persicae* and *Acyrthosiphon pisum*) (Troczka et al., 2015a; Dale et al., 2010).

Ryanodine receptors (RyRs) and inositol 1,4,5-trisphosphate receptors (IP_3_Rs) are large and complex calcium release channels; whilst RyRs are primarily located in the endo (sarco) plasmic reticulum of muscle cells, IP_3_Rs and RyRs are also found in many other cell types (Foskett et al., 2007; Hamilton and Serysheva, 2009). Both types of channels are inherently involved in the release of Ca^2+^ from internal stores - however, each channel has a distinct biological function; RyRs are involved in the regulation of calcium release during excitation-contraction coupling in muscle tissues and are primarily activated either by free Ca^2+^ or direct interaction with Ca_v_1.2 located in the plasma membrane (depending on channel isoform) (Fill and Copello, 2002), whereas IP_3_Rs are involved in complex spatio-temporal Ca^2+^ dynamics that have been implicated in a wide variety of biological functions from gene expression and apoptosis to learning and memory, and are primarily activated by the secondary messenger inositol 1,4,5-trisphosphate (IP_3_) (Foskett et al., 2007).

Partly due to the effectiveness and consequent commercial success of diamide insecticides, namely flubendiamide and chlorantraniliprole, which act as selective activators of insect RyRs (Cordova et al., 2006; Ebbinghaus-Kintscher et al., 2007), these channels have recently been receiving a considerable amount of attention from the scientific community, especially from researchers interested in understanding the mechanisms underlying the development of diamide resistance. Consequently, the number of RyR cDNAs being sequenced and cloned from a variety of agriculturally important pest species has rapidly increased (Nauen and Steinbach, 2016; Troczka et al., 2017). In comparison, IP_3_Rs remain significantly understudied in invertebrates. To date, only two insect IP_3_Rs have been cloned and experimentally tested; one from the *Drosophila melanogaster* (Yoshikawa et al., 1992) and the other from *Tribolium castaneum* (Liu et al., 2014). Functional expression of the *D. melanogaster* IP_3_R showed that its physiological properties are highly conserved in relation to its mammalian counterparts (Srikanth et al., 2004). The genomic organisation of IP_3_Rs however remains largely uncharacterised in insects - to date only *T. castaneum* IP_3_R has had its intron/exon organisation reported (Liu et al., 2014).

Although automated gene prediction tools are indispensable for genome annotations, these can occasionally generate incorrect gene structures (Yandell and Ence, 2012). The aim of this paper has therefore been to catalogue and validate a number of RyR and IP_3_R protein sequences, together with their genomic organisations, in 26 representative insect species, to help with future annotation of calcium release channels in genomic datasets and to further our understanding of the receptors' diversity in insects. Understanding the genomic structures of the insect channels could also contribute to a deeper appreciation of the evolution and regulation of these genes. IP_3_Rs are also candidate targets for the development of new classes of anti-insect molecules.

## 2 Materials and methods

### 2.1 In silico annotation and data mining

26 insect species from 5 insect orders with publicly available genomes (Diptera, Coleoptera, Hemiptera, Hymenoptera, Lepidoptera), 3 representatives of other insect orders (Blattellidae, Locusta, Phthiraptera) and one outlying acarine species (*Tetranychus urticae*) of the order Trombidiformes were included in the study (for a list of genome projects used for data generation see Appendix A). For each of the 5 main insect orders studied, a ‘reference species’ sequence was chosen to BLAST against the other available genomes within that particular order. The choice of ‘reference species’ sequence was based on a previous cloning and annotation of the sequence and the overall quality of the available genome. In the case of RyR the 5 reference species were: Dipteran = *D. melanogaster*, Hemipteran = *Myzus persicae*, Coleopteran = *T. castaneum*, Hymenopteran = *Apis mellifera*, Lepidopteran = *Bombyx mori*. For the IP_3_R, *D. melanogaster* and *T. castaneum* IP_3_Rs were the only sequences available to be used as a reference. PCR validation of Hymenopteran and Hemipteran IP_3_R genes was carried out to confirm the automated annotations. Three additional species, *Blattella germanica, Locusta migratoria* and *Pediculus humanus corporis*, were chosen for analysis based on the ‘completeness’ of their RyR genomic region, low contig fragmentation and few intra-contig gaps, determined by tblastn results against the WGS contig database with a reference RyR sequence.

Relevant contigs were downloaded from the NCBI database and manually curated using Geneious software v. 8.1.3 (Biomatters, Ltd., New Zealand). Translated exon sequences from the ‘reference species’ were blasted against databases created in Geneious using the tblastn algorithm. Megablast was used when DNA sequences were compared. Multiple sequence alignments were done using the MAFFT plugin in Geneious.

Splign (https://www.ncbi.nlm.nih.gov/sutils/splign/splign.cgi) (Kapustin et al., 2008) and manual curation was used to map intron-exon junctions, using as a curation guide the sequences of the most closely related reference species. Predicted RyR ORFs were extracted from the genomic sequence and validated with multiple alignments against a database of 25 manually curated insect and mammalian RyR sequences obtained from the NCBI database (see Appendix B). Gene structure graphs were generated using WebScipio (http://www.webscipio.org/) (Hatje et al., 2011). A web version of GenePainter (http://www.motorprotein.de/genepainter/genepainter) (Muhlhausen et al., 2015; Hammesfahr et al., 2013) was then used to generate intron phylogeny analysis based on the structure of the genes.

Structural features of analysed RyRs were determined based on multiple alignments of protein sequences with the previously annotated *P. xylostella* RyR (NCBI accession AET09964) (Troczka et al., 2017) and Pfam annotations (http://pfam.xfam.org/).

### 2.2 PCR validation of IP_3_R receptors

Total RNA from *M. persicae* and *B. terrestris* were extracted using Trizol reagent (Life technologies, CA, USA) or ISOLATE II RNA mini kit (Bioline, UK) following the manufacturer's guide. 4 μg of total RNA was used to synthesise cDNA in 20 μl reactions containing SuperScript® III Reverse Transcriptase (Thermo Fisher Scientific, USA) and Oligo _d_T (15) primers (Promega, USA), following the manufacturer's instructions. PCR primers were designed based on the in silico predicted sequences using Geneious software v. 8.1.3 (Biomatters, Ltd., New Zealand). PCR reactions were performed in 25 μl volumes using 2× Dreamtaq mastermix (ThermoFisher Scinetific, USA) with 10pmols of each primer and 1 μl of cDNA. Details of the PCR primers used can be found in Supplementary Tables 2 and 3. All PCR reactions were analysed on 1% (w/v) TAE agarose gels and visualised using ethidium bromide staining and UV light. PCR products were purified from the agarose gel using QIAquick gel extraction kit (Qiagen, Germany), following the manufacturer's recommended protocol, and directly sequenced using Eurofins Genomics Value Read service.

## 3 Results & discussion

### 3.1 Structural organisation of insect RyR's

**Fig. 1:**
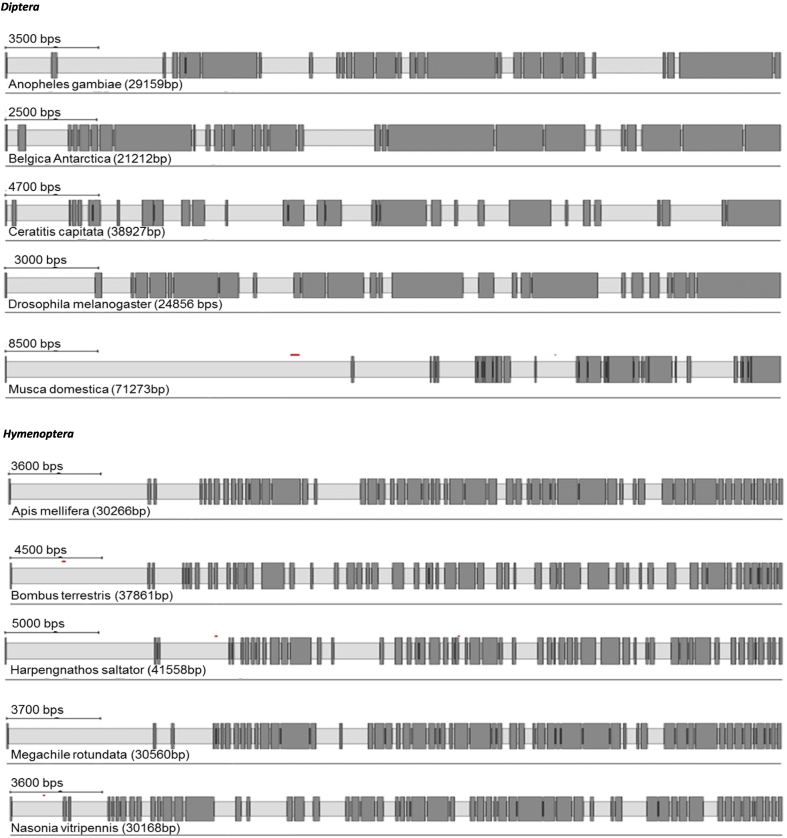

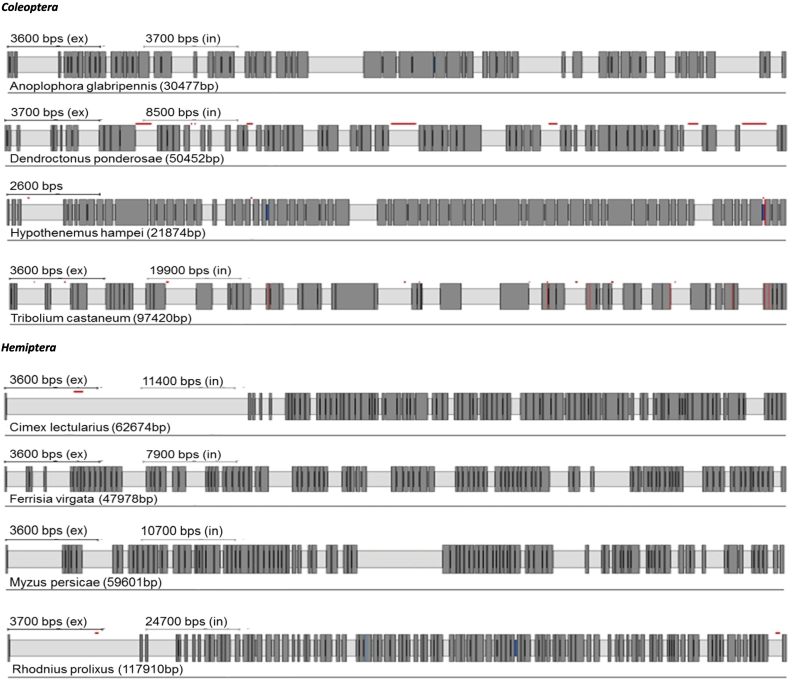

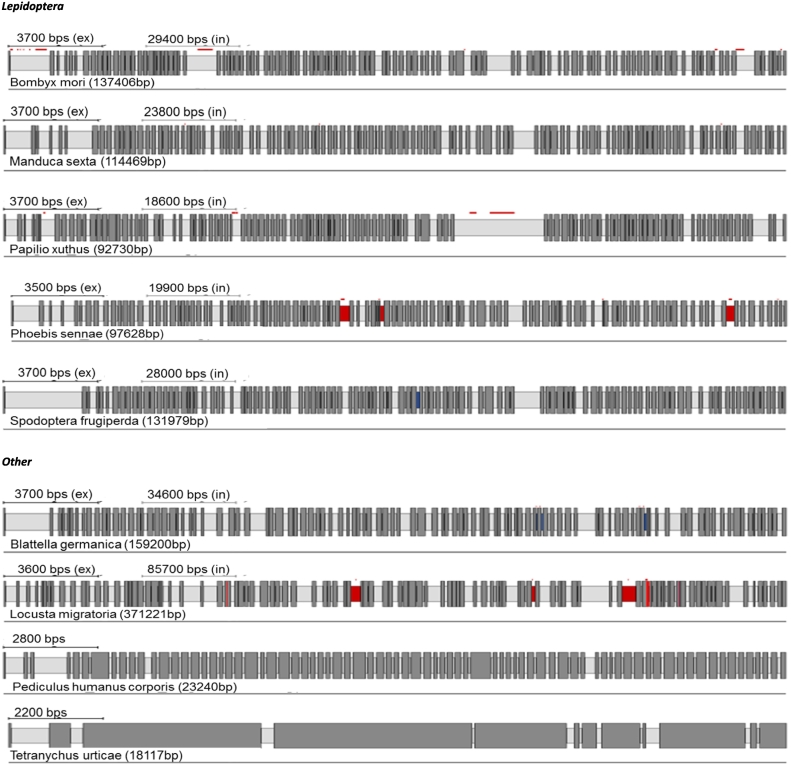
RyR gene structures generated by Webscipio. Dark grey regions correspond to exons. Red dashes indicate gaps in the sequencing, blue dashes indicate some uncertainty in intron assignment (non-canonical intron boundaries). (For interpretation of the references to colour in this figure legend, the reader is referred to the web version of this article.)

RyR cDNA sequences and their corresponding genomic organisation have been previously confirmed experimentally in *D. melanogaster, M. persicae*, *T. castaneum* and *B. mori* (Takeshima et al., 1994; Liu et al., 2014; Troczka et al., 2015a; Xu et al., 2000; Kato et al., 2009). Here we have manually annotated the RyRs of a further 23 insect species. A summary of the predicted channels and intron/exon arrangements of all 26 insect species can be found in Table 1 and Fig. 1. Additional information can be found in Supplementary excel file (SUP Table 1).

**Fig. 2:**
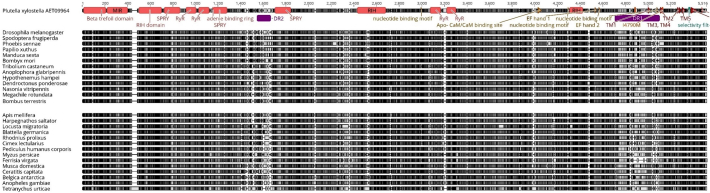
MAFFT alignment and Pfam domain mapping of annotated RyR sequences vs *P. xylostella* RyR. Red arrows indicate individual transmembrane helixes. (For interpretation of the references to colour in this figure legend, the reader is referred to the web version of this article.)

Protein alignments and Pfam database searches were performed to compare RyRs from different insect species. As expected, all the insect RyRs presented the classical cytosolic motifs and domains previously annotated in *P. xylostella* (Troczka et al., 2017) including one MIR (220–400), two SPRY domains, (726–862 and 1702–1841), four RYR domains (917–1007, 1031–1120, 3075–3166 and 3220–3303), three RIH domains (503–702, 2433–2682, 4307–4423) and EF calcium binding motifs (4531–4576). The numbering is based on a consensus sequence of a MAFFT alignment of all 27 RyR sequences with the annotated *P. xylostella* RyR (Fig. 2). The transmembrane (TM) region of the channel, which according to three-dimensional reconstructions using Cryo EM structures consist of six α-helixes (Yan et al., 2015; Zalk et al., 2015), was the most highly conserved region of the protein across all examined insect species.

**Table 1:** Summary of annotated sequences for ryanodine receptors.

Order	Species	Exon	Accession no.	Protein (AA)	Gene (bp)	Genome (Mb)
Diptera	*Anopheles gambiae* (African malaria mosquito)	29	XP_318561	5109	29,159	265.027
	*Ceratitis capitata* (Mediterranean fruit fly)	31	XP_012158404	5140	38,927	479.048
	***Drosophila melanogaster*** (**fruit fly**)	26	NP_001246211	5134	24,856	143.726
	*Musca domestica* (house fly)	30	XP_011296550	5127	71,276^a^	750.404
	*Belgica antarctica* (Antartic midge)	28		5088	21,215	89.5837
Hymenoptera	***Apis mellifera*** (**honey bee**)	54		5107	30,266	250.287
	*Bombus terrestris* (buff-tailed bumblebee)	54	XP_012175586	5108	37,861	248.654
	*Nasonia vitripennis* (jewel wasp)	56	XP_003425568	5099	30,168	295.781
	*Megachile rotundata* (alfalfa leafcutting bee)	55		5109	30,560	272.661
	*Harpegnathos saltator* (Jerdon's jumping ant)	54		5101	41,561	294.466
Coleoptera	***Tribolium castaneum*** (**red flour beetle**)	54	NP_001308588	5094	97,420	165.944
	*Dendroctonus ponderosae* (mountain pine beetle)	70		5137	50,615	252.848
	*Anoplophora glabripennis* (Asian longhorned beetle)	63		5091	30,477	707.712
	*Hypothenemus hampei* (coffee berry borer)	66		5107	21,874	151.272
Hemiptera	***Myzus persicae*** (**green peach aphid**)	98	AJA41114	5101	59,601	347.313
	*Cimex lectularius* (bed bug)	91	XP_014249567	5093	62,674	650.478
	*Rhodnius prolixus* (Assassin bug)	103		5103	117,911	706.824
	*Ferrisia virgata* (striped mealybug)	100		4982	47,978	304.571
Lepidoptera	***Bombyx mori*** (**domestic silkworm**)	110		5121	137,409	481.819
	*Papilio xuthus* (Asian swallowtail butterfly)	109		5124	92,730	243.89
	*Manduca sexta* (tobacco hornworm)	110		5127	114,469	419.424
	*Phoebis sennae*^a^ (cloudless sulphur butterfly)	108^a^		5109	97,628	287.49
	*Spodoptera frugiperda* (fall armyworm)	109		5127	131,979	358.048
Acari	*Tetranychus urticae* (two-spotted spider mite)	12	XP_015783312	5200	18,117	90.8286
Other orders	*Blattella germanica* (German cockroach)	103		5120	159,200	2037.2
	*Pediculus humanus corporis* (human body louse)	78	XP_002424547	5058	23,243	110.781
	*Locusta migratoria*^b^ (migratory locust)	103^b^		5130	371,221	5759.8

a The ORF in *Pheboes sennae*, although located on a single scaffold, remains incomplete as exons 48, 102, 103 and part of exon 54 are missing. This is likely due to gaps in the published scaffold sequence.

b The ORF in *Locusta migratoria* also remains incomplete with predicted exons 44, 68 and 78 being missing and sequencing gaps are present in exons 52, 58, 80 and 88. Due to missing sequence information the number of predicted exons for these two genes differs on manual curation compared with the webscipio/gene painter analysis.

The TM region of RyR is thought to be the binding site of diamide insecticides (Kato et al., 2009). In support of that hypothesis a glycine to glutamic acid substitution at position 4946 of the transmembrane domain of *Plutella xylostella* RyR has been shown to be a major cause of diamide resistance in this pest insect (Troczka et al., 2015b; Troczka et al., 2012; Steinbach et al., 2015). Recently, a novel glycine to valine substitution at this position has been identified in diamide resistant populations of *Tuta absoluta* (Roditakis et al., 2017). Additionally, three other mutations (E1338D, Q4594L and I4790M) have been associated with diamide resistant strains of *P. xylostella* (Guo et al., 2014). The equivalent of I4790M, located in the second TM helix and coming to close proximity to G4946 in a 3D model of insect RyR, was also found in some resistant populations of *T. absoluta* (Roditakis et al., 2017). Interestingly, the glycine residue at position 4946 appears to be conserved in most insect species, apart from *B. antarctica* and *T. utricae* which have an alanine in this position and *F. virgata* which has an arginine. It remains to be tested, however, whether these changes have any implications on diamide binding. Additionally, the neighboring amino acids in these later three species have a lower level of conservation in comparison to other insects (SUP Fig. 1). This amino acid residue “flexibility” within a highly conserved region could be one of the reasons diamide resistance mutations have emerged relatively quickly and without any apparent fitness costs (Ribeiro et al., 2014). In contrast to G4946 the isoleucine at position 4790 appears to be unique to lepidopterans, with all other insect species analysed in this study having a methionine or valine (found only in *F. virgata*). Therefore, it is hypothesised that an isoleucine at position 4790 may confer selectivity for some diamides towards lepidopteran insects. Q4594 is located in divergent region one with several different amino acids being found at this position, the most common being lysine (present in Coleoptera, Hymentoptera and some Diptera), glutamine (Lepidoptera), histidine (some Hemiptera), arginine (*A. gambiae*) and asparagine (*M. persicae*). E1338 is located between the first SPRY domain and divergent region two and like Q45954 shows little amino acid conservation with various amino acids (valine, glycine, serine, glutamine, aspartic acid, glutamine) found in different species.

### 3.2 Structural organisation of insect IP_3_R's

**Fig. 3:**
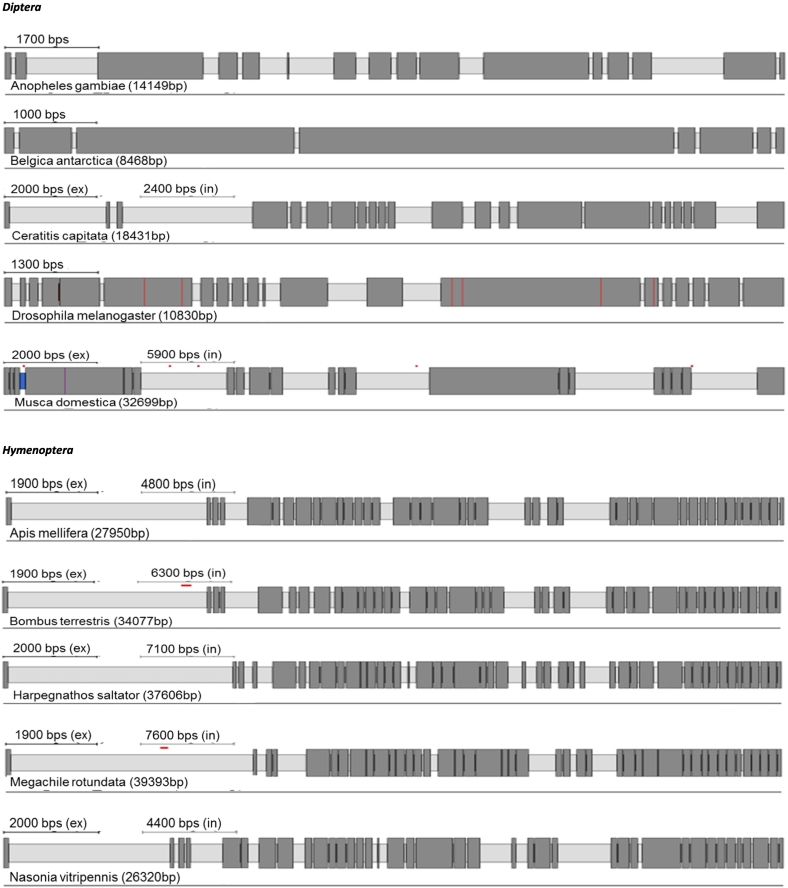

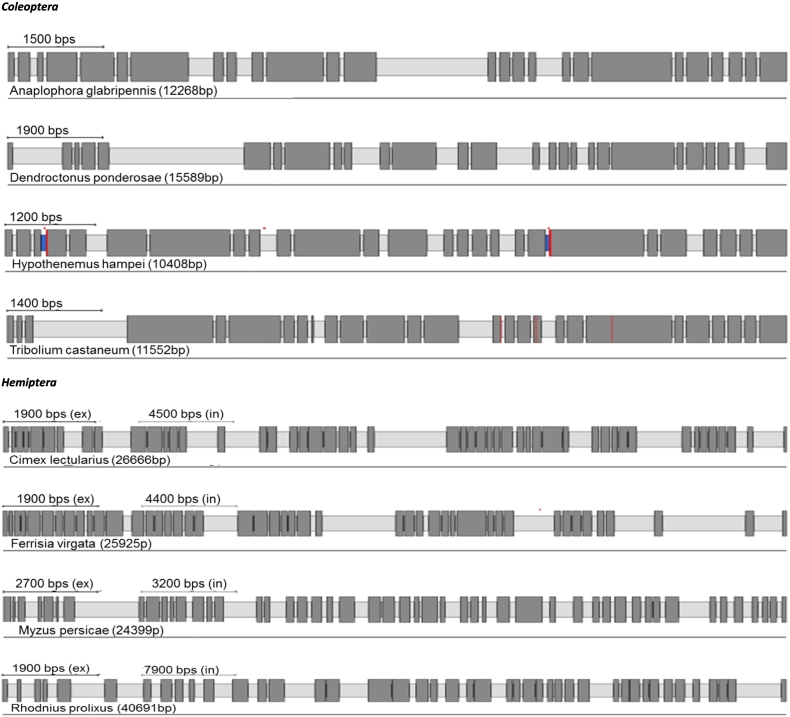

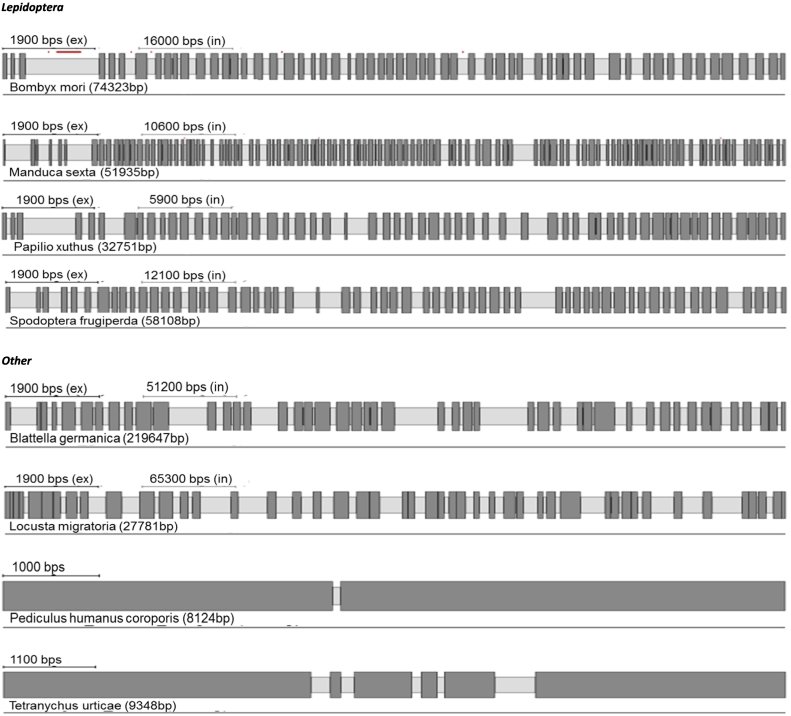
IP_3_R gene structures generated by Webscipio. Dark grey regions correspond to exons. Red dashes indicate sequence gaps, blue indicate some uncertainty in intron assignment (non-canonical intron boundaries). (For interpretation of the references to colour in this figure legend, the reader is referred to the web version of this article.)

IP_3_R cDNA sequences and their corresponding genomic organisation have been previously confirmed experimentally only in *D. melanogaster* and *T. castaneum* (Liu et al., 2014; Yoshikawa et al., 1992). Here we have manually annotated the IP_3_Rs of a further 24 insect species. A summary of the predicted channels and intron/exon arrangements of all 26 insect species can be found in Table 2 and Fig. 3.

**Table 2:** Summary of annotated sequences for inositol 1,4,5-triphosphate receptors.

Order	Species	Exon	Accession no.	Protein (AA)	Gene (bp)	Genome (Mb)
Diptera	*Anopheles gambiae* (African malaria mosquito)	16	XP_557157	2830	14,149	265.027
	*Ceratitis capitata* (Mediterranean fruit fly)	22	XP_012156517	2886	18,431	479.048
	***Drosophila melanogaster*** (**fruit fly**)	19	P29993	2838	10,830	143.726
	*Musca domestica* (house fly)	22		2800	32,699	750.404
	*Belgica antarctica* (Antartic midge)	8		2694	8468	89.5837
Hymenoptera	*Apis mellifera* (honey bee)	43		2727	27,950	250.287
	***Bombus terrestris*** (**buff-tailed bumblebee**)	43	XP_003394052	2727	34,077	248.654
	*Nasonia vitripennis* (jewel wasp)	38	XP_016839078	2745	26,320	295.781
	*Megachile rotundata* (alfalfa leafcutting bee)	42		2724	39,393	272.661
	*Harpegnathos saltator* (Jerdon's jumping ant)	44	XP_011151642	2741	37,606	294.466
Coleoptera	***Tribolium castaneum*** (**red flour beetle**)	27	NP_001308600	2724	11,552	165.944
	*Dendroctonus ponderosae* (mountain pine beetle)	27		2700	15,589	252.848
	*Anoplophora glabripennis* (Asian longhorned beetle)	26		2706	12,268	707.712
	*Hypothenemus hampei* (coffee berry borer)	26		2692	10,408	151.272
Hemiptera	***Myzus persicae*** (**green peach aphid**)	**49**		**3790**	**24,399**	**347.313**
	*Cimex lectularius* (bed bug)	49	XP_014243514	2735	26,666	650.478
	*Rhodnius prolixus* (assassin bug)	43		2634	40,691	706.824
	*Ferrisia virgata* (striped mealybug)	45		2645	25,926	304.571
Lepidoptera	***Bombyx mori*** (**domestic silkworm**)	58		2713	74,323	481.819
	*Papilio xuthus* (Asian swallowtail butterfly)	58		2722	32,751	243.89
	*Manduca sexta* (tobacco hornworm)	58		2717	51,935	419.424
	*Spodoptera frugiperda* (fall armyworm)	58		2707	58,102	358.048
Acari	*Tetranychus urticae* (two-spotted spider mite)	6	XP_015786315	2754	9348	90.8286
Other orders	*Blattella germanica* (German cockroach)	46		2661	219,647	2037.2
	*Pediculus humanus corporis* (human body louse)	2	XP_002425693	2680	8124	110.781
	*Locusta migratoria* (migratory locust)	46		2680	277,815	5759.8

**Fig. 4:**
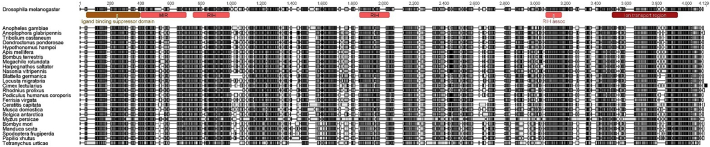
MAFFT alignment and Pfam domain map of annotated insect IP_3_R sequences.

A Pfam search of conserved domains for the IP_3_Rs identified 6 such domains across all 26 annotated channels. They include the IP_3_ binding region (44–398), MIR domain (402–703), two RIH domains (747–987, 1844–2031), RIH associated domain (3070–3177) and the transmembrane ion transport region (3508–3940). All numbering is based on the consensus sequence of the MAFFT alignment of the 26 annotated IP_3_Rs (Fig. 4). The transmembrane region of IP_3_R, as for RyRs, is thought to consist of 6 transmembrane helixes, which has been confirmed in a 3D structure of mammalian IP_3_R1 elucidated by Cryo-EM (Ludtke et al., 2011). Insect IP_3_Rs appear to have overall higher homology than RyRs to their mammalian counterparts with approximately 60% amino acid sequence identity (Srikanth et al., 2004).

Due to very little experimental information being available in the literature on cloning and sequencing of IP_3_R a decision was taken to PCR and sequence two of the insect IP_3_Rs channels, from *M. persicae* and *Bombus terrestris,* to validate our manual annotation predictions.

**Fig. 5:**
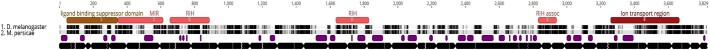
Alignment of *D. melanogaster* IP_3_R with *M. persicae* IP_3_R. Black arrows indicate individual exons. The approximate location of additional amino acids found in the aphid channel are indicated in purple. There are 36 individual insertions in the aphid channel with the largest being 63 amino acids in length. IP_3_R functional domains are mapped on the *D. melanogaster* sequence. (For interpretation of the references to colour in this figure legend, the reader is referred to the web version of this article.)

In silico annotation of *M. persicae* IP_3_R predicted a relatively larger channel compared to other insects. With a predicted ORF of 11,373 bp, *M. persicae* IP_3_R was projected to encode a 3790 aa protein, making it approximately 1000 aa larger than any other insect IP_3_R reported in this study, including those insects in the order hemiptera (e.g. *Bemisia tabaci* (Guo et al., 2017)). Protein alignment of the *M. persicae* channel *with D. melanogaster* IP_3_R indicated that the additional amino acids are scattered across the entire length of the protein (Fig. 5). Some of these insertions are present in functionally important domains, which could have a significant impact on the channels function. Interestingly the majority of insertions appear to be within the middle of the exons as opposed to 5′ or 3′ ends. Protein blast analysis of this predicted protein provided a good match up (over 90% identity) with other computationally predicted aphid IP_3_Rs, from pea aphid (*Acyrthosiphon pisum,* NCBI Acc. XP_001947344.2, encoding a 3831aa protein) and two predicted Russian wheat aphid alternatively spliced forms (*Diuraphis noxia*, NCBI Acc. XP_015373414.1 and XP_015373416.1, encoding proteins of 3816 and 3788 aa respectively). RT-PCR validation of the *in-silico* prediction for *M. persicae* IP_3_R confirmed that the receptor is much larger in this species in comparison to other insects, and that the predicted extra amino acids are present in the cDNA and are not intronic sequences. These larger IP_3_R channels appear to be unique to aphids. Pfam analysis of the protein sequence matches it with IP_3_R receptors, with conserved domains: Ins145_P3_rec (45–313), two MIR domains (345–476, 530–583), RIH (627–860) and the ion transport domain (3404–3624). The reason for a much larger IP_3_R in aphids is not apparent and we have found no evidence of similarly enhanced IP_3_Rs in other insect orders, so there is no clear evolutionary lineage. IP_3_R is the more ancient of the two channels studied (Alzayady et al., 2015). Evidence for larger IP_3_Rs-like channels (over 3000 amino acids) was reported in protozoan species such as *Paramecium* (Ladenburger and Plattner, 2011) and the filasterean *Capsaspora owczarzaki* (Alzayady et al., 2015); however these channels show very little conservation to the aphid protein. A significantly increased receptor size clearly has the potential to have a substantial impact on the channel's physiology and regulation. It has also been previously reported that aphids have an unusual heterodimeric voltage-gated cation channel, with close sequence homology with the voltage-gated sodium channel in other insects, albeit with an altered selectivity filter and being encoded by two unique heteromers (Amey et al., 2015; Zuo et al., 2016; Jiang et al., 2017). It is worth speculating that significant structural changes to at least two important ion channels could be indicative of a unique ion physiology in aphids in comparison to other insects.

Initial computational prediction of *B. terrestris* IP_3_R projected an ORF of 8370 bp (NCBI Acc. XP_012175773.1). However, RT-PCR validation followed by cDNA sequencing gave an ORF of 8184 bp (encoding a 2727 aa protein), which is a perfect match to another automatically predicted isoform of *B. terrestris* IP_3_Rs (NCBI Acc. XP_003394052). In comparison to the initial *in silico* prediction, the PCR validated sequence is missing two predicted exons (exons 23 and 28). We therefore assume that these missing exons are not part of the canonical channel, as we did not detect them in our sequenced PCR fragments. They might, however, represent rare transcripts specific to *B. terrestris* and other Hymenopterans, as BLAST results for both exons generate hits exclusively to species in this order.

The overall protein sequence similarity between different insect orders for the RyRs and IP_3_Rs was 80% and 70% respectively; the Hymenoptera was the least diverse order with over 90% identity among analysed species for both channels (93.5% for RyR and 92.29% for IP_3_Rs).

### 3.3 History of intron gain and loss within and across insect orders

There is a considerable variation in the number of introns present in RyR and IP_3_R genes across insect orders and other arthropods, reflected in the number of exons recorded for each species (Table 1, Table 2). However, there is generally only a small variation in the number of introns within each order, irrespectively of the genome size. For example, in Diptera, *Musca domestica* RyR has only two additional introns in comparison to *B. antarctica* despite its genome being 8.4 times larger (750 Mb and 89 Mb respectively). Substantial variation in intron number within a single order was only observed for Dipteran IP_3_R genes, with the flightless midge, *B. antarctica*, having only 7 introns compared with 21 introns for *M. domestica* and *C. capitata.* Species with the most complex IP_3_R belong to Lepidopterans (58 exons), followed by Hemipterans and Hymenopterans (over 40 exons) making them almost as complex as human IP_3_R genes with 63 exons for human type 1 and 2 isoforms (NCBI gene ID: 3708 and 3709). The RyRs of the representative species of the orders Diptera, Hymenoptera and Coleoptera showed significant changes in intron number in comparison to mammalian orthologues. For Lepidoptera, Hemiptera and Orthopteroida the total intron numbers recorded are close to the human isoforms (~105 introns) (Phillips et al., 1996).

**Fig. 6:**
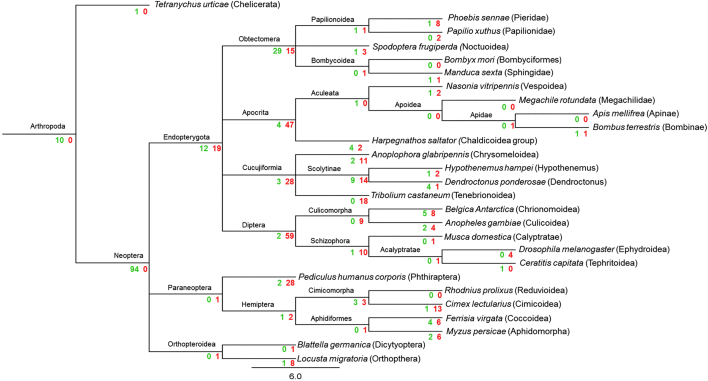
Phylogenetic distribution of intron positions for insect RyRs generated by GenePainter. The phylogenetic tree indicates those taxon branches at which introns were lost (coloured red) or gained (coloured green). (For interpretation of the references to colour in this figure legend, the reader is referred to the web version of this article.)

**Fig. 7:**
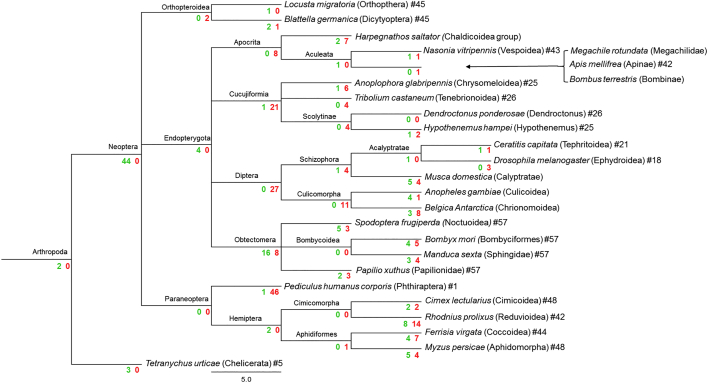
Phylogenetic distribution of intron positions for insect IP_3_R's generated by GenePainter. The phylogenetic tree indicates those taxon branches at which introns were lost (coloured red) or gained (coloured green). (For interpretation of the references to colour in this figure legend, the reader is referred to the web version of this article.)

A summary of loss and gain of introns across species is presented in the Genepainter phylogenetic ‘intron distribution’ trees for RyR and IP_3_Rs (Fig. 6, Fig. 7). Notably within the Paraneoptera, the entire IP_3_R gene of *P. humanus corporis* is made up of only 2 large exons. This dramatic reduction in intron number could be related to the ectoparasitic lifestyle of the species and small genome size (Sundberg and Pulkkinen, 2015). Within the Endopterygota, a significant intron loss has occurred within RyR genes in 3 of the 4 main orders (Diptera, Coleoptera, Hymenoptera, Lepidoptera), with almost 75% of ancestral introns being lost in some Dipteran species (e.g. *D. melanogaster* with 25 introns). Only Lepidoptera (Obtectomera) have maintained a relatively high ancestral intron number whilst simultaneously showing evidence of intron gain, making their RyR genes the most complex in all of the targeted insect species included in this study. They contain up to 110 exons on average, comparable in complexity to their mammalian counterparts (Phillips et al., 1996). The same pattern is observed for the IP_3_Rs genes, with the most complex architecture being found in Lepidoptera (58 exons), whilst the Diptera display the greatest overall reduction in intron number (25 introns) of all the insect orders studied (with the notable exception of *P. humanus corporis* as discussed above).

**Fig. 8:**
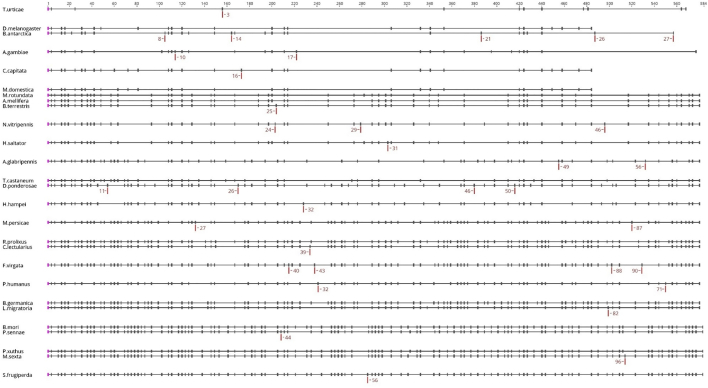
Gene structure alignment for insect RyRs obtained from Genepainter. Each dash corresponds to an intron overlaid on a protein alignment. To improve clarity the size of each exon is not representative of the actual exon size. Red dashes indicate unique introns and their number in individual species. (For interpretation of the references to colour in this figure legend, the reader is referred to the web version of this article.)

**Fig. 9:**
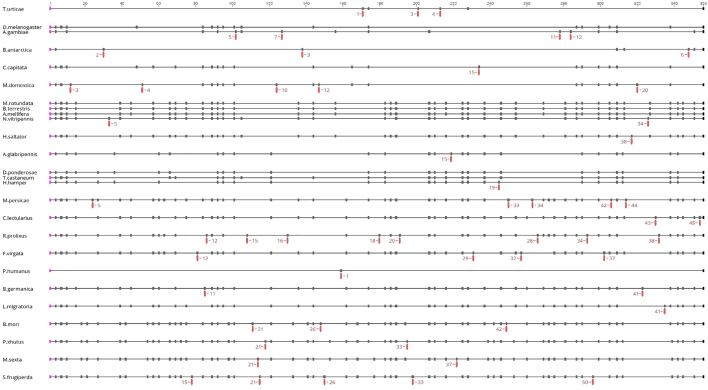
Gene structure alignment for IP_3_Rs obtained from Genepainter. Each dash corresponds to an intron. To improve clarity the size of each exon is not representative of the actual exon size. Red dashes indicate unique introns and their number in individual species. In total there are 56 unique introns mapped on the alignment. (For interpretation of the references to colour in this figure legend, the reader is referred to the web version of this article.)

Despite an overall high level of conservation at the protein (amino acid) level for both RyR and IP_3_R channels in insects, an extensive remodelling of the genomic structure has resulted in a low conservation of common introns across insect orders. GenePainter analysis showed only 8 common introns conserved within RyR across the insect species looked at in this study. This number falls to only 3 when *T. urticae* (Acari) is included in the analysis. Interestingly, 4 of the common (conserved) introns are located within the first 10 introns, with a further 2 encompassing a highly-conserved exon (exon 20 in *D. melanogaster*, 39 in *A. mellifera*, and *B. mori,* 71 in *M. persicae*). Looking at the overall distribution of intron positions, it appears that the greatest number of intron losses occur towards the 3′ end of the ORF (see SUP Table 1), possibly indicating the involvement of reverse transcriptase in this evolutionary process (Cohen et al., 2012). The conservation of intron-exon organisation does not appear to be linked with any particular structural features. All of the conserved intron-exon junctions occur within the cytosolic portion of the protein. The highest level of conservation is found in the introns surrounding a mutually exclusive splice site in the second SPRY domain, with *M. persicae* being the outlier. The second highest conserved pair of introns is found around the predicted calmodulin/Apo-calmodulin interaction site, which also appears to be alternatively spliced in some species. In contrast, there are 34 unique introns detected across all species (Fig. 8). There are no conserved introns for IP_3_Rs and only 2 conserved introns if *T. urticae* and *P. humanus corporis* are excluded. However, there are 56 unique introns found across the 26-species studied (Fig. 9). In comparison to the RyRs, the IP_3_R gene structure is less well conserved with a much higher proportion of unique introns in relation to the total intron number.

### 3.4 Alternative splicing of RyR and IP_3_R

Fully annotated gene structures can elucidate further useful information such as the regulatory mechanisms governing gene expression and the probability of alternative splicing (Chorev and Carmel, 2012). Understanding splicing regulation is a difficult challenge as the spliceosome is one of the most complicated molecular complexes, consisting of over 150 proteins (Wahl et al., 2009). Point mutations in the genomic structure may lead to modulation of the splicing machinery resulting in exon skipping (Cartegni et al., 2002). Such an event in the nicotinic acetylcholine receptor has already been linked with resistance to the insecticide spinosad in *Tuta absoluta* (Berger et al., 2016). Intron size and number have also been shown to directly correspond to splicing diversity (Chorev and Carmel, 2012; Fox-Walsh et al., 2005).

**Fig. 10:**
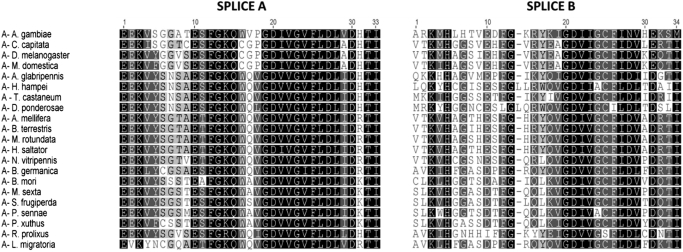
Multiple alignment of a mutually exclusive splice site found in 21 of the analysed insect RyR sequences.

RyRs are known to possess an inherently complex genomic organisation and several alternative spliced isoforms have been reported in insects (Xu et al., 2000; X. Wang et al., 2012; Puente et al., 2000). A common (mutually exclusive) alternative splice site has been found in 21 of the 26 studied species, the exceptions being *P. humanus*, *T. urticae*, *B. antarctia, C. lectularius* and the previously reported *M. persicae* (Troczka et al., 2015a). This site is located within the second SPRY domain in the N-terminal part of the channel (Fig. 10). SPRY domains are found in many mammalian proteins and are thought to be linked with immune responses (D'Cruz et al., 2013). In mammalian RyR1, the second SPRY domain is a site of interaction with the II-III loops of Ca_v_1.1 and also with scorpion toxin A (Tae et al., 2009). Although insect RyRs are thought not to be directly linked with Ca_v_1.1 channels (Takekura and Franzini-Armstrong, 2002), alternative exons in this region might play important roles in modulating the interaction of insect RyRs with other regulatory proteins. To date alternative exons have been best described in Lepidopteran species (Wang et al., 2013; J. Wang et al., 2012; Sun et al., 2015; Wu et al., 2013; Cui et al., 2013). As highlighted in Fig. 8, exon version A is present in all species lacking an alternative exon. Thus, version A is likely to be the most common splice form. In *M. persicae* this exon is fused with a neighboring exon indicating a clear intron loss event (Troczka et al., 2015a).

Apart from mutually exclusive exons, there is growing evidence, especially in Lepidoptera, of a number of deletions and insertions which result in a greater diversity of detectable splice forms in comparison to other insect species, as shown by the extensive splice forms detected in *P. xylostella* (X. Wang et al., 2012).

We did not attempt to map mutually exclusive exons in insect IP_3_Rs due to the scarcity of experimentally obtained mRNA sequences. However, due to this paucity of validated cDNA's the existence of such exons cannot be dismissed. Alternative splicing of IP_3_Rs is well documented in mammals (Foskett et al., 2007) and at least one of these splice sites appears to be conserved in *D. melanogaster* (Sorrentino et al., 2000).

## 4 Conclusions

Despite an ever-growing number of insect genomes becoming publicly available the quality of many of them remains problematic. Short contig lengths and a high degree of fragmentation make it challenging to fully annotate large genes such as RyRs and IP_3_Rs (Mackrill, 2012). Additionally, automatic annotations can omit certain sections of the gene (usually the 1st exon) if it is positioned a long distance from the rest of the gene and no reference transcriptome data is available. Manual curation of important genes and gene families is required to verify and improve genomic data. In the case of insects, validation of automatically annotated genes has not been done for many species. Certain insect orders remain over represented in the wet biology due to their importance to agriculture or disease control. Our study shows that despite a relatively high protein homology, the gene architecture of proteins can differ substantially among different insect orders. We have shown that there is a substantial variation in exon number and overall gene size for both RyR and IP_3_R channels and very little intron conservation across different species. Structural variation of genes coding for highly conserved proteins is likely to contribute to a great diversity in potential mRNAs among insects allowing for the existence of species and order-specific splice variants.

The following are the supplementary data related to this article.

SUP Table 1 Summary of exon assignments. SUP Table 1

Supplementary Tables 1,2,3, Supplementary Figure 1 Image 1

## Conflicts of interest

The authors declare no conflict of interest. The funding sponsors had no role in the design of the study; in the collection, analyses, or interpretation of data; in the writing of the manuscript, and in the decision to publish the results.

## Appendix A List of genome projects and Accessions used for data generation with associated peer reviewed publications (where appropriate)

*Tetranychus urticae* (BioProjects: PRJNA315122, PRJEA71041) (Grbic et al., 2011), *Drosophila melanogaster* (chromosome 2R ref. seq: NT_033778.4) (Hoskins et al., 2007), *Anopheles gambiae* (chromosome 3L ref. seq: NT_078267.5) (Holt et al., 2002), *Ceratitis capitata* (BioProjects: PRJNA201381, PRJNA168120) (Papanicolaou et al., 2016), *Belgica antarctica* (BioProject: PRJNA172148) (Kelley et al., 2014), *Musca domestica* (BioProject: PRJNA210139) (Scott et al., 2014), *Megachile rotundata* (BioProjects: PRJNA87021, PRJNA66515), *Apis mellifera* (Linkage group: NC_007071.3, BioProject: PRJNA10625) (Honeybee Genome Sequencing Consortium, 2006), *Bombus terrestris* (BioProjects: PRJNA68545, PRJNA45869) (Sadd et al., 2015), *Nasonia vitripennis* (BioProjects: PRJNA20073, PRJNA13660) (Werren et al., 2010), *Harpegnathos saltator* (BioProjects: PRJNA273397, PRJNA50203) (Bonasio et al., 2010), *Tribolium castaneum* (BioProjects: PRJNA15718, PRJNA12540) (Tribolium Genome Sequencing Consortium, 2008), *Dendroctonus ponderosae* (BioProject: PRJNA162621) (Keeling et al., 2013), *Anoplophora glabripennis* (BioProjects: PRJNA348318, PRJNA167479), *Hypothenemus hampei* (BioProject: PRJNA279497) (Vega et al., 2015), *Myzus persicae* (BioProjects: PRJNA397782, PRJNA319804), *Cimex lectularius* (BioProject: PRJNA167477) (Rosenfeld et al., 2016), *Rhodnius prolixus* (BioProject: PRJNA13648) (Mesquita et al., 2015), *Ferrisia virgata* (BioProject: PRJEB12067), *Blattella germanica* (BioProject: PRJNA203136), *Locusta migratoria* (BioProject: PRJNA185471) (Wang et al., 2014), *Pediculus humanus corporis* (BioProjects: PRJNA19807, PRJNA16223) (Kirkness et al., 2010), *Bombyx mori* (BioProject: PRJDA20217) (International Silkworm Genome Consortium, 2008), *Papilio xuthus* (BioProject: PRJDB2956), *Manduca sexta* (BioProject: PRJNA81037) (Kanost et al., 2016), *Spodoptera frugiperda* (BioProject: PRJNA257248) (Kakumani et al., 2014), *Phoebis sennae* (BioProject: PRJNA308118) (Cong et al., 2016).

## Appendix B Ryanodine receptor sequences (and associated NCBI Accession numbers) used for the validation alignments

*Aphis citricidus* (AKM95171.1), *Atta colombica* (KYM79740.1), *Bemisia tabaci* (AFK84957.1), *Cnaphalocrocis medinalis* (AFI80904.1), *Cerapachys biroi* (EZA52107.1), *Carposina sasakii* (AHN16453.1), *Chilo suppressalis* (AFN70719.1), *Daphnia pulex* (EFX89429.1), *Dialeurodes citri* (AKM95170.1), *Helicoverpa armigera* (AHB33498.1), *Homo sapiens* RyR1 (AAC51191.1), *Homo sapiens* RyR2 (CAA66975.1), *Homo sapiens* RyR3 (CAA04798.1), *Laodelphax striatella* (AFK84959.1), *Leptinotarsa decemlineata* (AHW99830), *Lucilia cuprina* (KNC23059.1), *Melipona quadrifasciata* (KOX73585.1), *Nilaparvata lugens* (KF306296.1), *Ostrinia furnacalis* (AGH68757.1), *Pieris rapae* (AGI62938.1), *Plutella xylostella* (AET09964.1), *Sogatella furcifera* (AIA23859), *Spodoptera exigua* (AFC36359.1), *Trachymyrmex cornetzi* (KYN11971.1), *Tuta absoluta* (APC65631.1).
