# Supplementary material for: An analysis of variability in genome organisation of intracellular calcium release channels across insect orders
Source: Gene. 2018 Sep 5;670:70–86. doi: 10.1016/j.gene.2018.05.075 (PMC6026295; doi:10.1016/j.gene.2018.05.075)
Supplement: Supplementary file 2 — Supplementary Tables 1,2,3, Supplementary Figure 1 [file mmc2.docx]

**Supplementary information**

**SUP Table 1.** Excel spreadsheet summary of exon assignment / alignment and intron and exon sequences for RyRs and IP_3_Rs. It should be noted that in some species, due to scaffold fragmentation and a large intron size, the final annotation is distributed across several contigs with some gaps in intronic sequence still remaining.

**SUP Table 2.** *Bombus terrestris* IP_3_R Primers

| **Primer name** | **Sequence** | **Start** | **End** |
| --- | --- | --- | --- |
| **BB IP3R Start f** | GAGATCCTCGGCTCCGCG | 7 | 24 |
| **BB IP3R Start R** | GGCGTTCACTGGTTCCAAAATCAC | 547 | 570 |
| **BB IP3R A f** | ACATCGTCAGCCTATACGCCG | 44 | 64 |
| **BB IP3R A r** | ACCTGACGAACGATGATTGTGGG | 1,110 | 1,132 |
| **Exon 7 gap F** | TGATTTTGGAACCAGTGAACGCC | 548 | 570 |
| **BB IP3R B f** | GATCCGACTACATTAACGAGAGGC | 1,075 | 1,098 |
| **BB IP3R B r** | CGGGCGTATTTAACTGGTGTCAC | 2,377 | 2,399 |
| **Exon 13 Gap F** | AGAAAAACATATCACGGCAGCGG | 1,794 | 1,816 |
| **BB IP3R C f** | CGCGCCTCATTTTGTCGACTTAT | 2,317 | 2,339 |
| **BB IP3R C r** | ACCGTATGTACTCCGACGTTACG | 3,619 | 3,641 |
| **BB IP3R D f** | AGCTCCGCCTCAATTGTCTACAT | 3,429 | 3,451 |
| **BB IP3R D r** | TCTTTGGTTGTTTCGCAGCTTGA | 4,812 | 4,834 |
| **BB IP3R E f** | AGACGTAGCTAAAGGAAGAGGCA | 4,704 | 4,726 |
| **BB IP3R E r** | ACATGAGCGTTTCGGATACCAGA | 6,093 | 6,115 |
| **BB IP3R F f** | AATGTGCGTAATCTTGCTTCCGG | 5,839 | 5,861 |
| **BB IP3R F r** | ACCGGATTCTCGAGGTAAGGTAA | 7,094 | 7,116 |
| **BB IP3R G f** | TATCTGCTCTTATCTGGGCGGTG | 7,046 | 7,068 |
| **BB IP3R G r** | CGGATGCAGAATTCAAAAGGCCT | 8,322 | 8,344 |
| **Exon 37 gap R** | ATAACAAGAGAATCGCATGCCCG | 7,699 | 7,721 |
| **BB IP3R END F** | CGGGCATGCGATTCTCTTGTTAT | 7,699 | 7,721 |

**SUP Table 3.** *Myzus persicae* IP_3_R Primers

| **Primer name** | **Sequence** | **Start** | **End** |
| --- | --- | --- | --- |
| **Mz. IP3 1F** | TAAGATGATGATTTCACCAGGAG | 1 | 19 |
| **Mz. IP3 1R** | ACAAAGTACACAATGTCCTGAAGCAGC | 1,881 | 1,907 |
| **Mz. IP3 2F** | CAACGATGCATGTAAAGTATTGG | 1,791 | 1,813 |
| **Mz. IP3 2R** | TCAATAGCGTTTTGGTGAGTCTGAGC | 3,348 | 3,373 |
| **Mz. IP3 3F** | AGTTCTACCATAGCATTCGTTGAGG | 3,196 | 3,220 |
| **Mz. IP3 3R** | AGACCATCCATACGTCGGTCTCC | 4,894 | 4,916 |
| **Mz. IP3 4F** | TGTAGGCGTACATACAGTAGTG | 4,839 | 4,860 |
| **Mz. IP3 4R** | TAATAGTGATAGTTCAGCTTGGACC | 6,669 | 6,693 |
| **Mz. IP3 5b F** | GGAAAACACAATACGCACACTT | 6,354 | 6,375 |
| **Mz. IP3 6b R** | TAAGCTCGACATGCCACATC | 8,869 | 8,888 |
| **Mz. IP3 6F** | CCCTGCCATGACAATCAAAATTGT | 8,641 | 8,664 |
| **Mz. IP3 6R** | AGCCGAAGCCAAAATCTGACCCAGC | 10,224 | 10,248 |
| **Mz. IP3 7F** | ACATTATTAGTATCATGGGCAATCAGG | 10,181 | 10,207 |
| **Mz. IP3 7R** | ACTCTACCGCCTCCACCAACGCC | 11,311 | 11,333 |

**
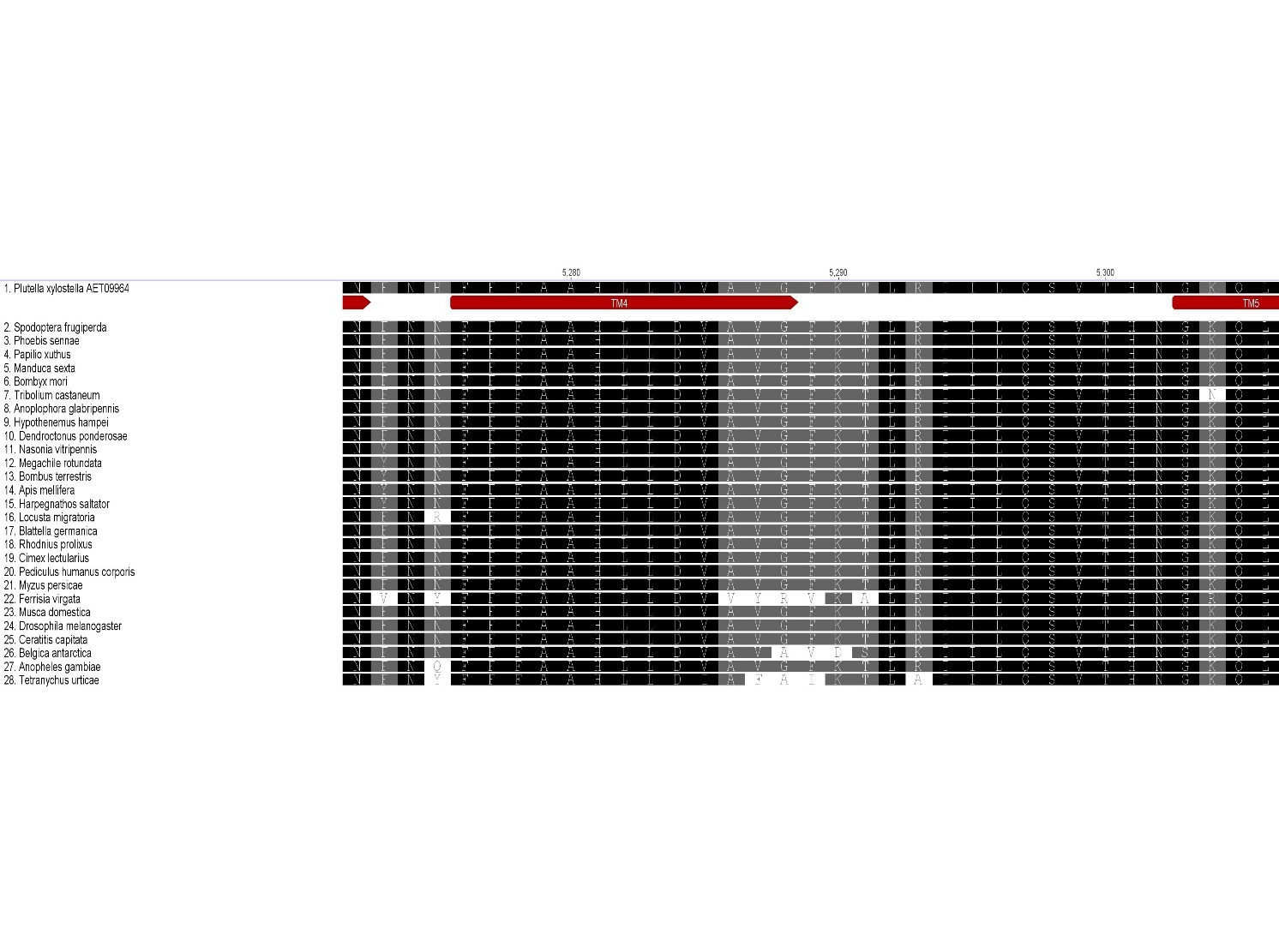
**

**SUP Figure 1.** G4946 (*P. xylostella* numbering) is conserved in most species except *F. virgata*, *B. antarctica* and *T. urticae*.
